# Evaluation of Different Doses of the Aromatase Inhibitor Letrozole for the Treatment of Ectopic Pregnancy and Its Effect on Villous Trophoblastic Tissue

**DOI:** 10.1007/s43032-022-00993-0

**Published:** 2022-06-14

**Authors:** Mohamed Ali Alabiad, Warda M. M. Said, Abdalla Hassan Gad, Mustafa Taha Abdelfattah Sharaf ElDin, Dina Ahmed Khairy, Mai Ahmed Gobran, Amany Mohamed Shalaby, Walaa Samy, Ahmed Ahmed Abdelsameea, Ahmed Ismail Heraiz

**Affiliations:** 1 Department of Pathology, Faculty of Medicine, Zagazig University, Zagazig, 44519 Egypt; 2 Department of Pathology, Faculty of Medicine, Benghazi University, Benghazi, Libya; 3 Department of Gynecology and Obstetrics, Faculty of Medicine, Zagazig University, Zagazig, Egypt; 4 Department of Biomedical Dental Sciences, College of Dentistry, Imam Abdulrahman Bin Faisal University, Dammam, Saudi Arabia; 5 Department of Histology and Cell Biology, Faculty of Medicine, Tanta University, Tanta, Egypt; 6 Department of Medical Biochemistry and Molecular Biology, Faculty of Medicine, Zagazig University, Zagazig, Egypt; 7 Department of Pharmacology, Faculty of Medicine, Zagazig University, Zagazig, Egypt

**Keywords:** Letrozole, Ectopic pregnancy, Induced abortion, Trophoblastic tissue ER, PR, VEGF, Cleaved caspase-3

## Abstract

Letrozole, an aromatase inhibitor, has recently been introduced as a favorable medical treatment for ectopic pregnancy. We aimed at evaluating the effects of different doses of letrozole for termination of ectopic pregnancy and study their effects on villous trophoblastic tissue. Sixty patients with undisturbed ectopic pregnancy were classified into three equal groups. Group I: the control group that contained women who underwent laparoscopic salpingectomy, Group II: patients who received letrozole (5 mg day^−1^) for 10 days, and Group III: patients who received letrozole (10 mg day^−1^) for 10 days. Subsequently, the β-hCG levels were determined on the first day and after 11 days of treatment. Group IV consisted of patients of GII and GIII; their β-hCG did not drop below 100 mIU/ml within 11 days, and underwent salpingectomy. Placental tissues from patients undergoing salpingectomy either from the control group or GIV were processed for the evaluation of estrogen (ER) and progesterone (PR) receptors, vascular endothelial growth factor (VEGF), and cleaved caspase 3 (CC-3) expression. Cases exposed to high dose letrozole 10 mg day^−1^ resulted in a higher ectopic pregnancy resolution rate of 85% (17/20), while the resolution rate of the low dose letrozole-treated group (5 mg day^−1^) was 65% (13/20), and also showed a significant reduction in β-hCG levels on the 11th day, 25.63 ± 4.29 compared to the low dose letrozole group 37.91 ± 7.18 (*P* < 0.001), Meanwhile, the letrozole-treated group GIV showed markedly reduced expression of ER, PR, and VEGF and a significant increase in the apoptotic index cleaved caspase-3 compared to the control group (*P* < 0.001). The utilization of letrozole at a dose of 10 mg day^−1^ for medical treatment of ectopic pregnancy results in a high-successful rate without any severe side effects. Letrozole depriving the placenta of estrogen that had vascular supporting signals resulted in destroying the vascular network with marked apoptosis.

**Graphical abstract:**

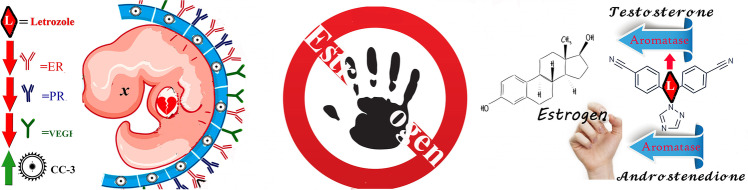

## Introduction

Ectopic pregnancy is the implantation and growth of the embryo outside the uterine cavity, which accounts for maternal morbidity and mortality [1]. Ectopic pregnancy affects around 1–2% of all pregnancies [2]. Although ectopic pregnancy–related mortality was recently reduced, ruptured ectopic pregnancies represent approximately 6% of all maternal deaths [3]. Although some ectopic pregnancies resolve spontaneously and need only conservative treatment, others continue to grow and lead to tubal rupture that results in severe or persistent abdominal pain and life-threatening intraabdominal hemorrhage that required further interference. Medical and surgical treatment are two methods for the management of growing ectopic pregnancy [4, 5]. Recently, medical treatment has been used as the first choice to treat the early discovered ectopic pregnancy. While using methotrexate as a medical treatment for ectopic pregnancy was associated with terrible adverse effects on different body systems, as well as high proportions of failure in ectopic pregnancy termination [6]. Therefore, it is necessary to find more cost-effective and safe alternatives. Letrozole, a non-steroidal competitive aromatase inhibitor, is approved for the management of estrogen-sensitive breast cancer. Letrozole inhibits estrogen production from androgen aromatization via inhibition of the electron transfer chain of cytochrome P450 (CYP19A1) [7]. Letrozole is also used in the treatment of many gynecological disorders, mainly due to its high tolerability, low cost, and low side effects, including the reduction of the size of uterine myomas [8, 9], induction of ovulation in cases of polycystic ovarian syndrome [9], and the reduction of endometriosis recurrence [10–12]. In humans, letrozole inhibits estrogen production during pregnancy and negatively affects placental, estrogen, progesterone, and VEGF expression [6, 13], with subsequent disruption of progesterone physiological function, which was required to support early pregnancy [14], and the interruption of VEGF vascular support signals, which is the angiogenic factor involved in implantation and placentation of ectopic pregnancy [15], which drew attention to how letrozole hinders pregnancy progression [6].

Therefore, our objective was to evaluate different doses of letrozole for the treatment of ectopic pregnancy to reach the optimal and most safe dose with the highest success rate of ectopic pregnancy termination, and also, to investigate the possible underlying mechanisms through determining ER and PR expression, apoptosis signaling molecules, and their consequence on placental angiogenesis.

## Patients and Methods

This study included 60 patients with undisturbed ectopic pregnancy admitted to the Department of Obstetrics and Gynecology of the Faculty of Medicine of Zagazig University from October 2020 to July 2021. Inclusion criteria include patients with confirmed ectopic pregnancy on vaginal ultrasound examination, together with β-hCG titers < 3000 mIU/ml, between 19 and 34 years old, with asymptomatic and hemodynamically stable pregnancy. Exclusion criteria included β-hCG levels > 3000 mIU/ml, hemoglobin level < 10 g/dl, platelet count < 150,000/ml, and elevated liver enzymes, blood urea, or serum creatinine. The patients were equally categorized into three groups: patients who were undergoing laparoscopic salpingectomy (the control group GI), patients who were medically treated with 5 mg day^−1^ of letrozole using two tablets (2.5 mg of Femara) every day for 10 days (group II), and patients who medically treated with 10 mg day^−1^ of letrozole using four tablets (2.5 mg of Femara) every day for 10 days (group III). Meanwhile, each woman selected her treatment, and all patients had no contraindications to letrozole treatment. The β-hCG levels, platelet count, liver enzymes, and serum creatinine were assessed on the 1st and 11th days of treatment. Patients who had no successful response to medical treatment (β-hCG levels had not decreased below 100 mIU/ml on the 11th day after treatment with letrozole) either from group II or III had to undergo salpingectomy and formed group IV. Patients with a β-hCG level below 100 mIU/ml on the 11th day of treatment divided by the total number of patients in the group refer to the success rate of abortion.

Tissue samples from laparoscopic salpingectomy either from control group GI or group IV were collected and fixed with 10% buffered formal saline and impeded in paraffin blocks [16], and processed for estrogen (ER), and progesterone (PR) isoforms, using a real-time PCR (RT-PCR) as previously described [17–19], and vascular endothelial growth factor (VEGF), and cleaved caspase-3 were detected by immunohistochemistry.

The Institutional Review Board of Zagazig University Hospital authorized this study with an approval number of ZU-IRB/19/N/106/13, and all patients gave their informed written consent.

### Immunohistochemical Study

Immunohistochemical staining for VEGF in a 1/20 dilution (a mouse monoclonal antibody (JH121) Catalog # MA5-13,182, Invitrogen, Thermo Fisher Scientific, USA) and cleaved caspase-3 1/50 dilution (a recombinant Rabbit Monoclonal Antibody (9H19L2), Catalog # 700,182, Invitrogen, Thermo Fisher Scientific, USA). Briefly, Paraffin blocks were cut into 4 μm cuts. Immunostaining was performed using an autostainer from Leica BOND-MAXTM (Leica GmbH, Nussloch, Germany), using the manufacturer’s guidelines [20]. The slides were dewaxed in BondTM dewax solution (Leica Microsystems) and rehydrated in Leica Microsystems (Bond Wash Solution). Antigen retrieval at pH 6 was done via Bond Epitope Retrieval 1 Solution (Leica Microsystems) at 100 °C for 30 min. Slides were incubated with monoclonal primary antibodies against (VEGF, and cleaved caspase-3) for 20 min at RT. Biotin off-bond polymer refines recognition (Leica Microsystems) was used to visualize the primary antibody interacting with tissue slices [21]. After post-primary amplification and recognition with the Novolink polymer revealing apparatus, the slides were counterstained with hematoxylin (Leica Microsystems). Lastly, the slides were dried, cleared, and fixed by DPX.

### Morphometric Analysis

A Leica light microscope (DM500, Switzerland) was used in conjunction with a Leica digital camera (ICC50, Switzerland) to obtain the images. Software “ImageJ” (version 1.48v National Institute of Health, Bethesda, Maryland, USA) was utilized for image testing [22, 23]. A 10 dissimilar non-overlying randomly chosen fields were observed from each slide to quantitatively estimate the percentage of positive cells for cleaved caspase-3 with a × 40 objective [24], and for VEGF scoring by multiplying the mean percentages of positive cells and the intensity of the color, color intensity was measured as 1, 2, and 3 for weak, moderate, and strong intensity, respectively. [25]

### Quantitative Real-Time PCR

Total RNA was extracted using Trizol reagent (Invitrogen), according to the manufacturer’s protocol. The total RNA concentration was measured using a spectrophotometer. First-strand complementary DNA (cDNA) was prepared from total RNA (3 μg) by reverse transcription (RT) using M-MLV reverse transcriptase (Invitrogen) and random primers (9-mers; TaKaRa Bio). Quantitative real-time PCR (Q-PCR) was performed with a cDNA template (2 μL), and 2 × Power SYBR Green (6 μL; TOYOBO Co., Osaka, Japan) containing specific primers (β-actin, ERα, Erβ, PR) are given as follows:

**Table:** 

Gene name	Primer	Sequence (5′–3′)	Fragment (bp)
β-actin	Forward	GGACTTCGAGCAAGAGATGG	234
	Reverse	AGCACTGTGTTGGCGTACAG	
ER α	Forward	AGCACCCTGAAGTCTCTGGA	153
	Reverse	GATGTGGGAGAGGATGAGGA	
ER β	Forward	AAGAAGATTCCCGGCTTTGT	173
	Reverse	TCTACGCATTTCCCCTCATC	
PP14	Forward	GACCAACAACATCTCCCTCAT	170
	Reverse	AAACGGCACGGCTCTFCCATC	
PR	Forward	GGCGGATCCGTCAAGTGGTCTAAATCATTG	351
	Reverse	GGCGAATTCCTGGGTTTGACTTCGTAGCCC	

PCR was carried out for 40 cycles using the following parameters: denaturation at 95 °C for 15 s, followed by annealing and extension at 70 °C for 60 s. The fluorescence intensity was measured at the end of the extension phase of each cycle. The threshold value for the fluorescence intensity of all samples was manually set. The reaction cycle at which PCR products exceeded this fluorescence intensity threshold during the exponential phase of PCR amplification was the threshold cycle (CT). The expression of the target gene was quantified relative to that of β-actin, a ubiquitous housekeeping gene, based on a comparison of CTs with constant fluorescence intensity.

### Statistical Analysis

Data were analyzed using SPSS software (25.0, SPSS Inc. Chicago, IL, USA) and were described as means and standard deviations. One-way ANOVA followed by a post hoc test was used to assess the significance among means, with *P* < 0.05 as the significant level.

## Results

In this study, a total of 60 patients with undisturbed ectopic pregnancy were divided into three groups. The first group (G1) was subjected to laparoscopic salpingectomy and served as the control group, while the second and third groups (GII and GIII) were subjected to medical treatments with 5 and 10 mg day^−1^ of letrozole, respectively. The demographic and clinical characteristics of all patients were presented in Table 1. No significant differences were found between the three groups concerning the age, body mass index, parity, β-hCG levels, platelet count, ALT, AST, and creatinine levels on the day of treatment.

**Table 1:** Demographic and clinical characteristics differences between the three clinical groups

		Group type									ANOVA	Multiple comparisons		
		Group I (*n* = 20)			Group II (*n* = 20)			Group III (*n* = 20)			*P*-value	I and II	I and III	II and III
Age (years)	Mean ± SD	24.45	±	3.13	26.85	±	4.14	24.85	±	2.70	0.064			
	Range	19	−	30	21	−	34	20	−	29				
BMI, kg/m^2^	Mean ± SD	21.01	±	2.021	20.53	±	1.93	22.03	±	2.19	0.664			
	Range	17.9	−	24.5	18.4	−	25	18	−	24.4				
Parity	Mean ± SD	0.99	±	0.66	1.31	±	0.59	1.06	±	0.52	0.207			
	Range	0	−	2.1	0	−	2.1	0	−	2.1				
B-HCG treatment day	Mean ± SD	1192.60	±	193.89	1186.10	±	168.71	1173.75	±	118.42	0.860			
	Range	797	−	1601	1000	−	1395	1015	−	1355				
B-HCG 11th day	Mean ± SD	22.43	±	7.67	37.91	±	7.18	25.63	±	4.29	< 0.001	< 0.001	0.279	< 0.001
	Range	8.9	−	36.6	25.4	−	49.8	17.4	−	31.1				
Success abortion rate	Percent	20/20		100%	13/20		65%	17/20		85%	0.012	0.008	0.231	0.144
AST level (U/L) Treatment day	Mean ± SD	19.50	±	2.69	20.67	±	2.35	19.25	±	2.46	0.173			
	Range	14.9	−	24.3	17	−	24.8	15.2	−	24.1				
AST level (U/L) Day 11	Mean ± SD	20.63	±	2.59	24.29	±	2.61	49.090	±	4.146	< 0.001	0.002	< 0.001	< 0.001
	Range	15.7	−	25.4	19.6	−	28.8	39.6	−	55.8				
ALT level (U/L) Treatment day	Mean ± SD	28.80	±	1.52	29.029	±	1.72	28.68	±	1.84	0.6048			
	Range	26.12	−	30.98	26.23	−	32.87	26.00	−	32.76				
ALT level (U/L) Day 11	Mean ± SD	29.92	±		28.86	±	3.68	54.68	±	10.86	< 0.001	0.883	< 0.001	< 0.001
	Range	19.9	−	39.8	19.8	−	38.8	26.9	−	63.9				
S. creatinine (mg/dl) treatment day	Mean ± SD	0.70	±	0.05	0.61	±	0.22	0.69	±	0.09	0.08			
	Range	0.61	−	0.79	0.37	−	0.89	0.56	−	0.80				
S. creatinine (mg/dl) day 11	Mean ± SD	0.62	±	0.08	0.54	±	0.20	0.58	±	0.07	0.10			
	Range	0.50	−	0.71	0.33	−	0.79	0.47	−	0.69				
Platelets (× 10^3^/µL) treatment day	Mean ± SD	228	±	62	256	±	55	219	±	67	0.13			
	Range	161	−	298	189	−	323	121	−	296				
Platelets (× 10^3^/µL) Day 11	Mean ± SD	207	±	59	222	±	45	217	±	59	0.37			
	Range	132	−	281	163	−	283	140	−	298				

The resolution rate of ectopic pregnancy in the control group GI was 20/20 (100%) of patients compared to 13/20 (65%) of patients in low-dose letrozole-treated group GII and 17/20 (85%) in the high-dose treated group GIII. Ten patients who had no successful response to medical treatment within 11 days of either GII or GIII had to undergo salpingectomy and form GIV.

### Biochemical Results

There is no statistically significant difference between the three groups concerning β-hCG levels, platelet count, S. creatinine, ALT, and AST, on the treatment day was found; also, there is no statistically significant difference between the three groups concerning platelet count and S. creatinine on the 11th day of treatment.

While high-dose letrozole-treated group GIII exhibited a significant decrease in β-hCG levels on the 11th day of treatment 25.63 ± 4.29 compared to low-dose letrozole group GII 37.91 ± 7.18 (*P* < 0.001), also high-dose letrozole-treated group GIII exhibited a significant increment in the serum ALT and AST levels (54.680 ± 10.86 and 49.09 ± 4.14 U/L) when compared with the control group (29.92 ± 4.33 and 20.63 ± 2.59 U/L), respectively (*P* < 0.001) (Table 1).

### Immunohistochemistry Results

**Fig. 1:**
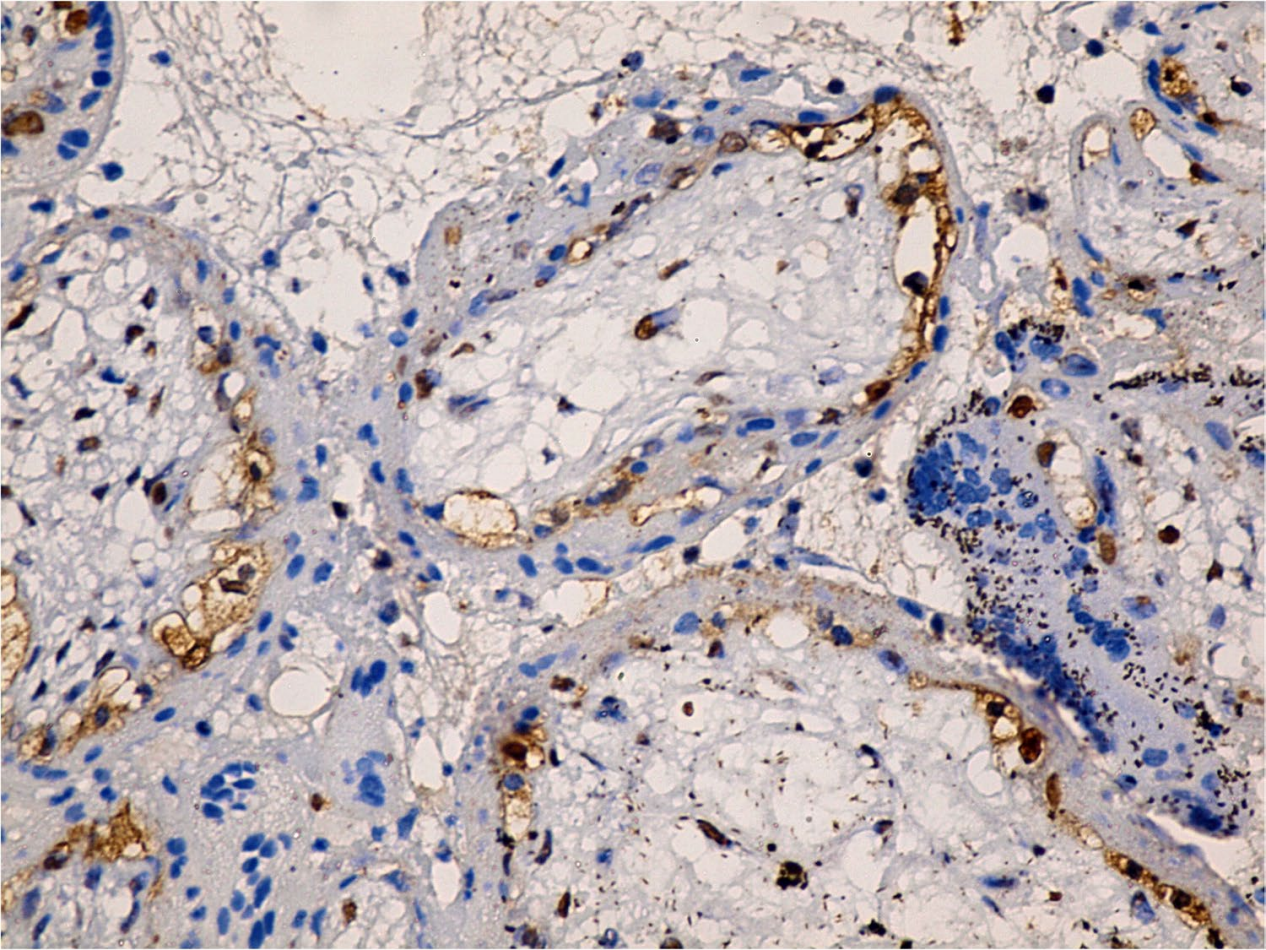
Immunohistochemistry of a few cleaved caspase-3 positive cells in villous trophoblastic cells of the control group (400 ×)

**Fig. 2:**
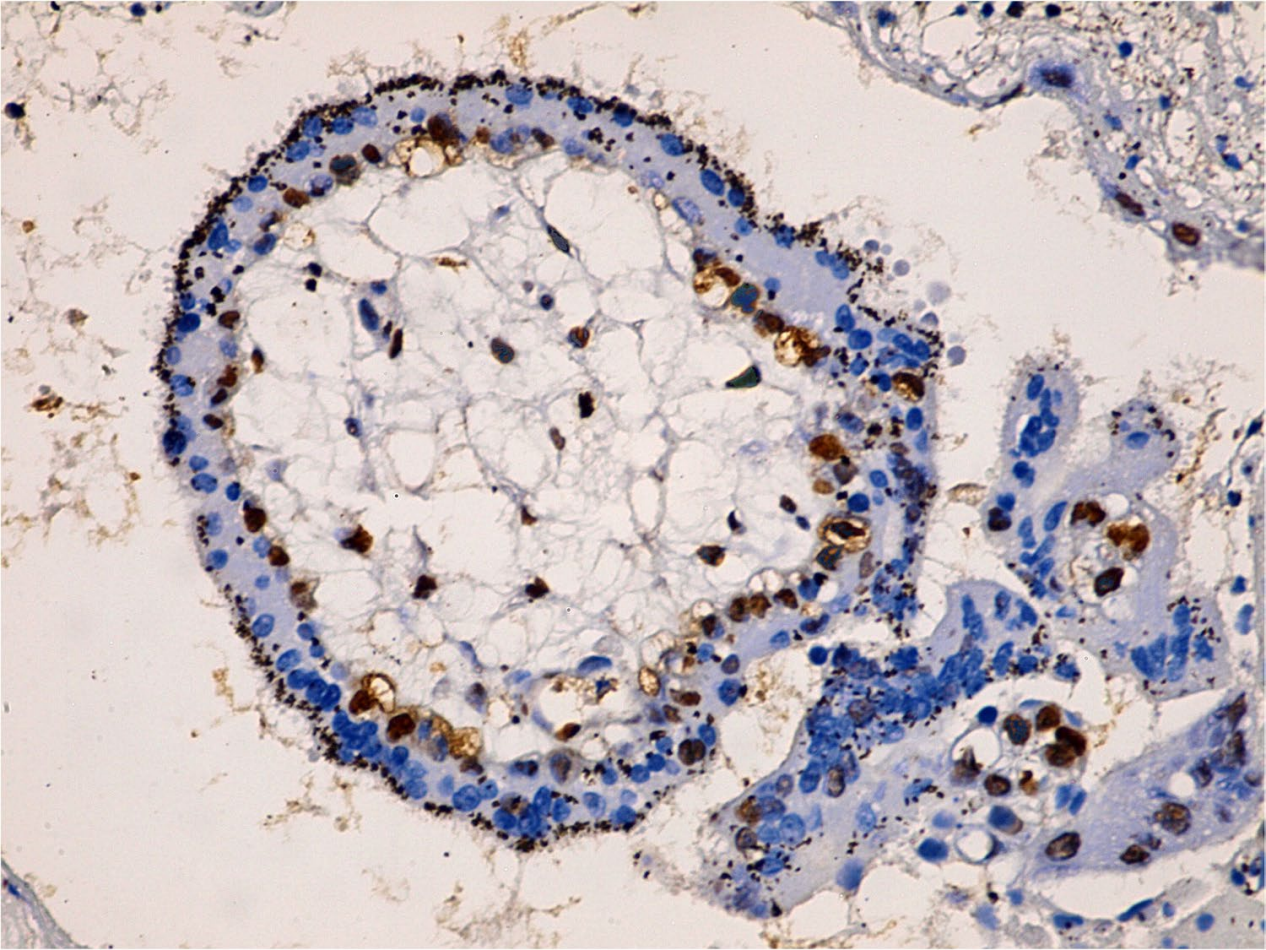
Immunohistochemistry of few cleaved caspase-3 positive cells in outer trophoblasts and inner villi mesenchymal core cells of the control group (400 ×)

**Fig. 3:**
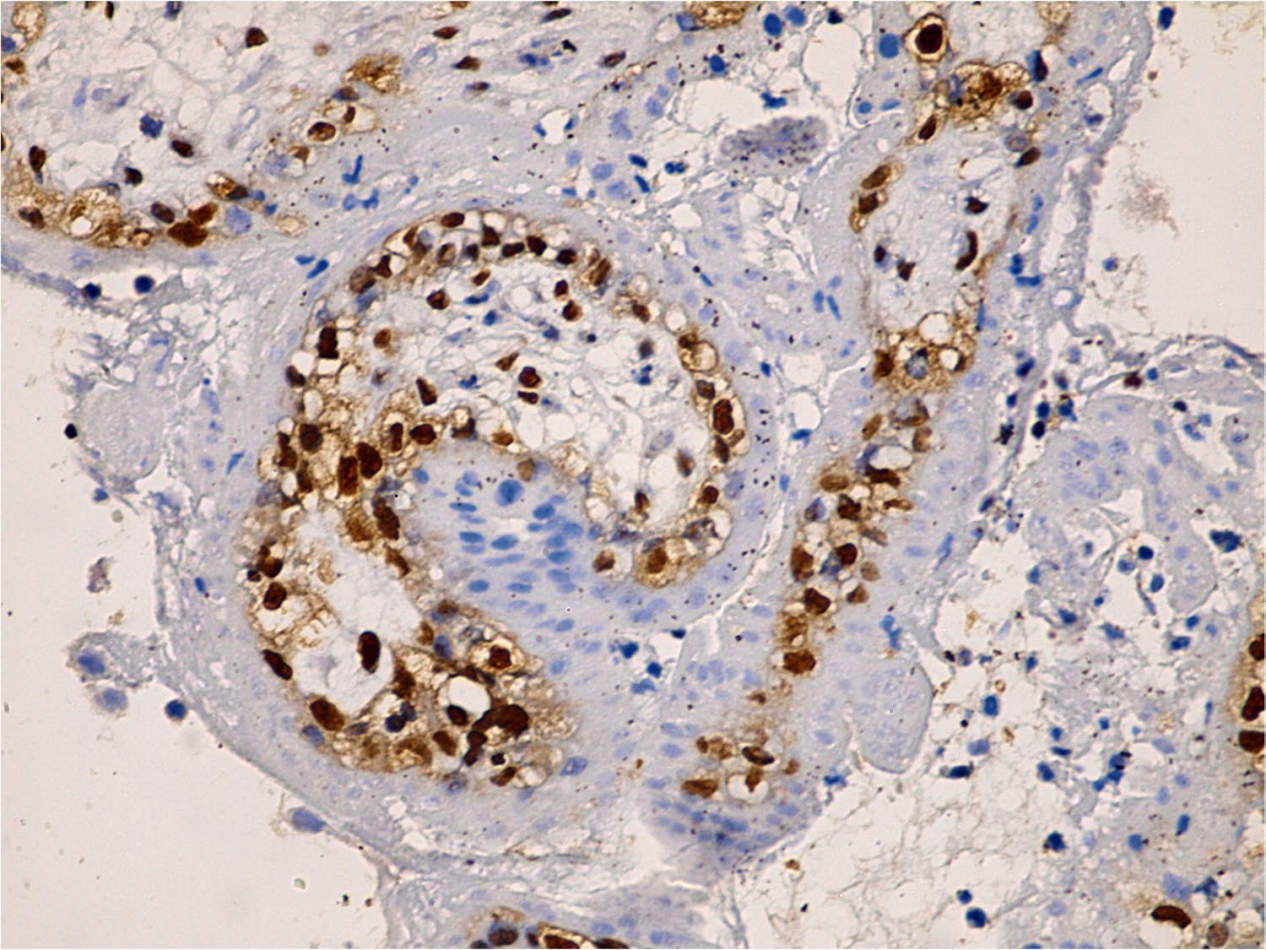
Immunohistochemistry of numerous cleaved caspase-3 positive cells in the outer trophoblastic cells of the letrozole-treated group IV (400 ×)

**Fig. 4:**
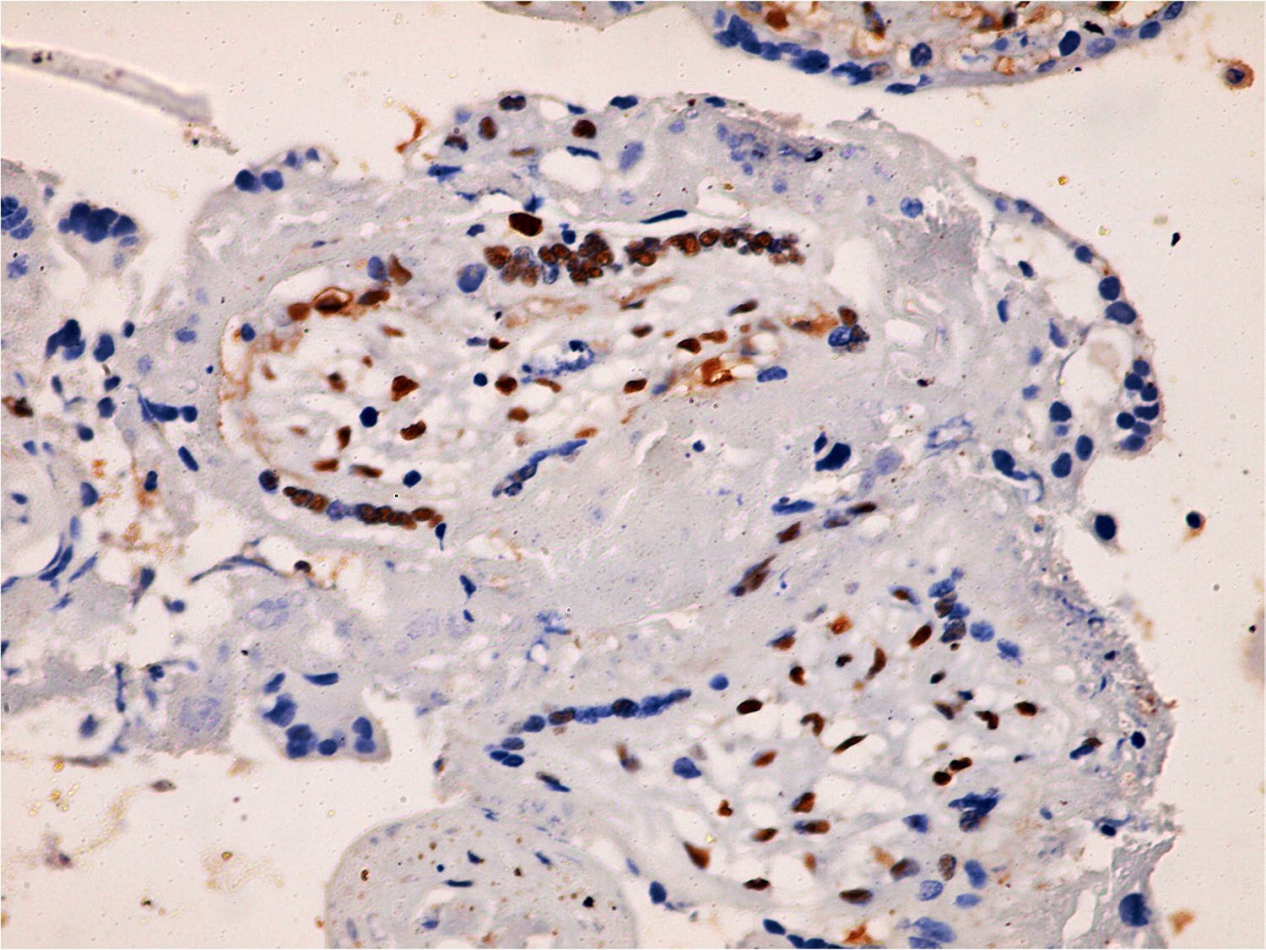
Immunohistochemistry of numerous cleaved caspase-3 positive cells in the inner villi's mesenchymal core cells of the letrozole-treated group IV (400 ×)

The sections of the control group had low expression of cleaved caspase-3 (Figs. 1 and 2), compared to the GIV group treated with letrozole, which demonstrated numerous positive cells (Figs. 3 and 4). The morphometric examination of the mean percentage of cleaved caspase-3 positive cells of the control group GI showed (18 ± 2, SD), which was significantly decreased than the letrozole-treated group GIV (38 ± 4) (*P* < 0.001) (Table 2).

**Table 2:** The effects of exposure to letrozole on trophoblastic tissue of control and letrozole-treated groups

		Group type						
		Group I (*n* = 20)			Group IV (*n* = 10)			*P*-value
ER α	Mean ± SD	0.5	±	0.1	0.2	±	0.1	< 0.001*
	Range	0.3	−	0.6	0.1	−	0.4	
ER β	Mean ± SD	0.6	−	0.1	0.3	−	0.1	< 0.001*
	Range	0.4	±	0.8	0.1		0.4	
PR	Mean ± SD	0.9		0.2	0.4	±	0.2	< 0.001*
	Range	0.6	−	1.2	0.2	−	0.7	
Caspase-3C	Mean ± SD	18	±	2	38	±	4	< 0.001*
	Range	15	−	26	32	−	47	
VEGF	Mean ± SD	121.27	±	9.1	64.31	±	4.37	< 0.001*
	Range	102.34	−	145.26	56.64	−	73.23	

**Fig. 5:**
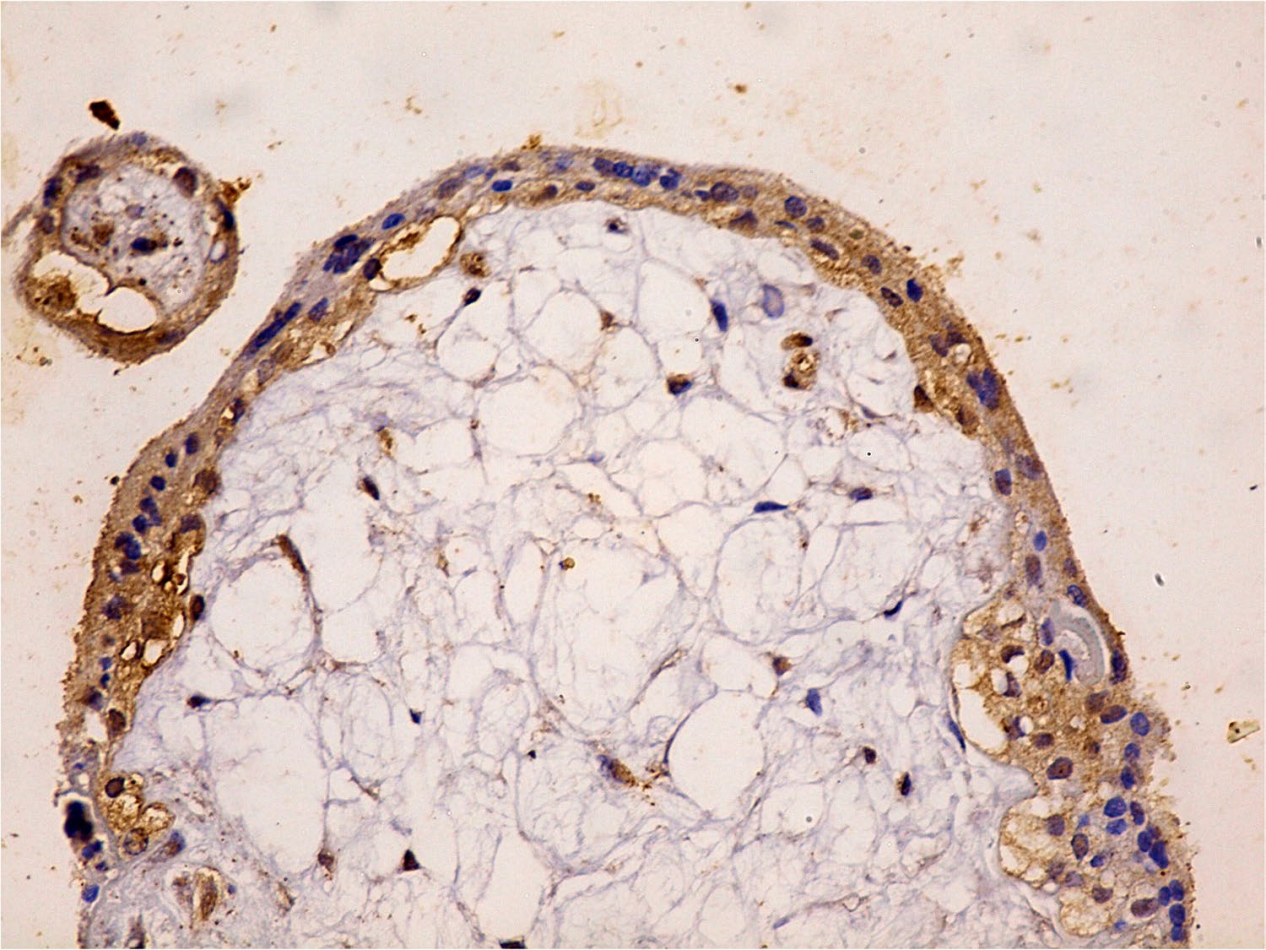
Immunohistochemistry of strong expression of VEGF in outer trophoblastic cells of the control group villi (400 ×)

**Fig. 6:**
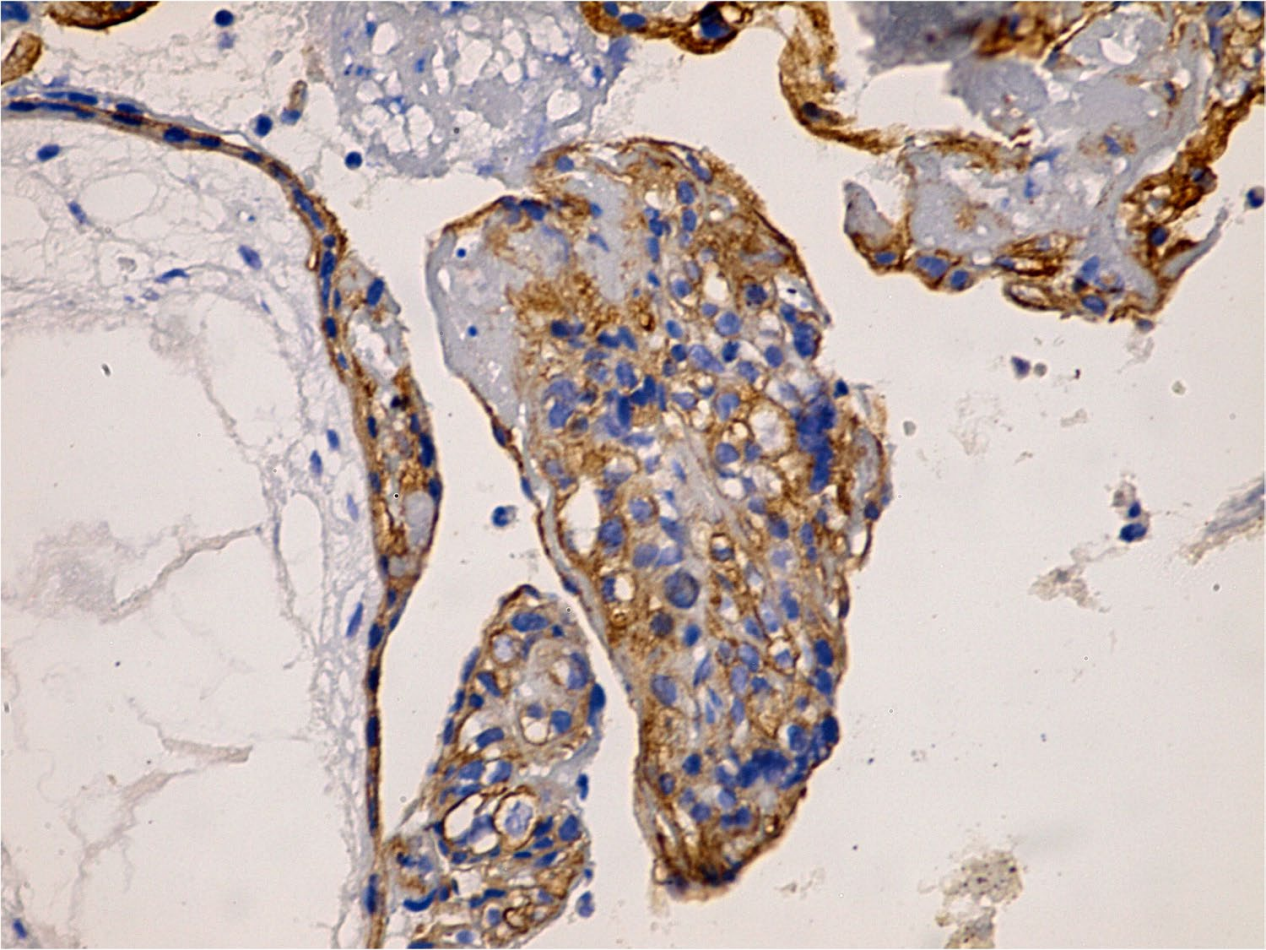
Immunohistochemistry of strong VEGF expression in inner villi mesenchymal core cells of inner villi control group (400 ×)

**Fig. 7:**
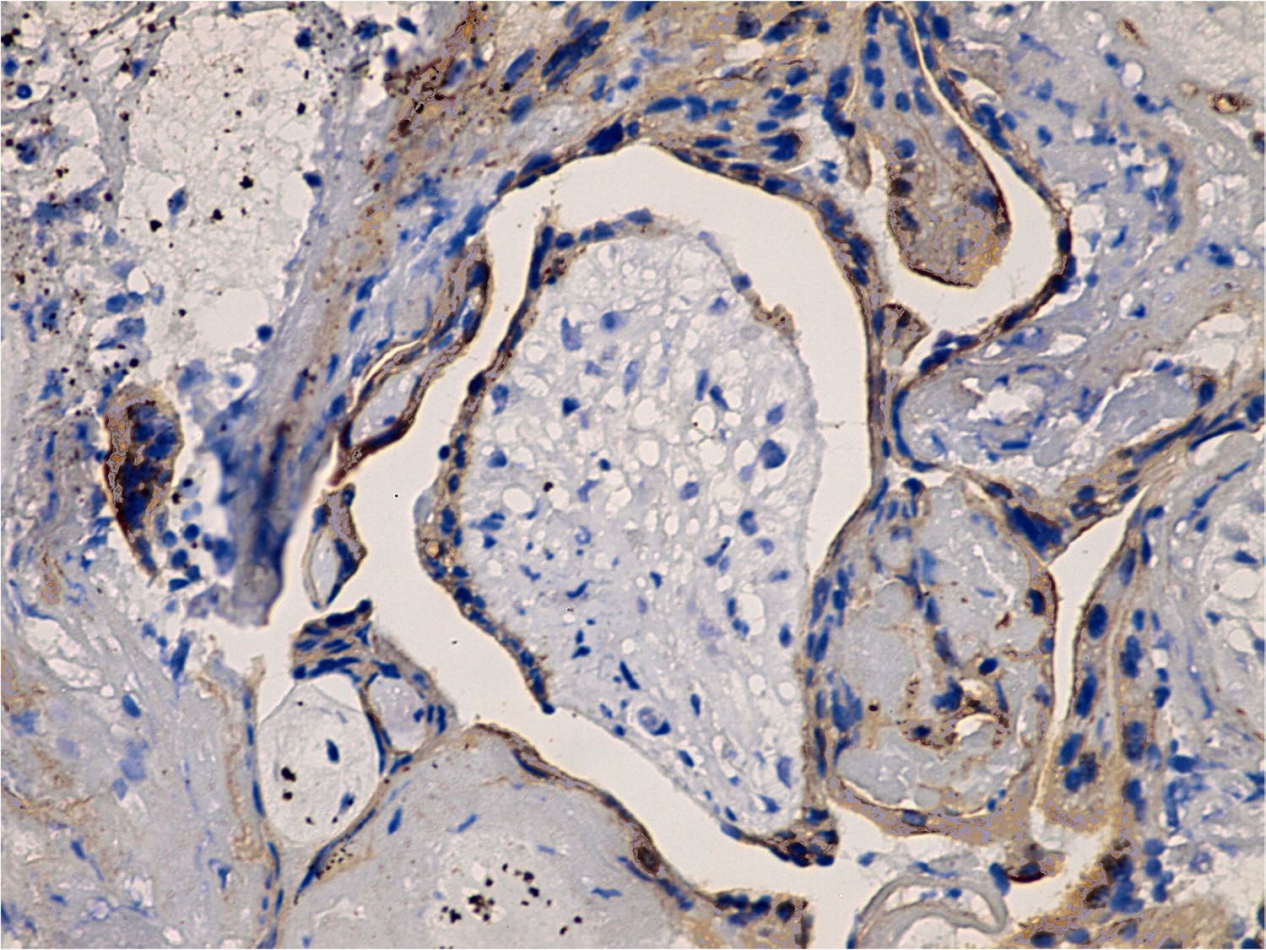
Immunohistochemistry of weak VEGF expression in outer trophoblasts and inner core cells of the villi of the letrozole-treated group IV (400 ×)

Placental tissues immunolabelled with VEGF from the control group exhibited strong cytoplasmic expression in villous syncytiotrophoblast, cytotrophoblast, Hofbauer cells of the stroma, and internal fetal capillaries (Figs. 5 and 6). Meanwhile, the letrozole-treated group showed focal areas with weak expression of cytoplasmic VEGF (Fig. 7). The morphometrical and statistical investigation of the final score of VEGF expression in the letrozole-treated group GIV showed an exceedingly significant decrease in the expression (64. 31 ± 4. 37) compared to the control group GI (121.27 ± 9.1) (*P* < 0.001) (Table 2).

### Quantitative Real-Time PCR Results:

In this study, the RT-qPCR results showed a markedly significant decrease in the levels of ERα, Erβ, and PR mRNA expression levels of the letrozole-treated group GIV (0.2 ± 0.1, 0.3 ± 0.1, and 0.4 ± 0.2), respectively, compared to the control group (0.5 ± 0.1, 0.6 ± 0.1, and 0.9 ± 0.2) (*P* < 0.001).

## Discussion

Ectopic pregnancy is extrauterine implantation usually in the fallopian tube; some ectopic pregnancies resolve spontaneously, but others continue to grow and lead to rupture of the tube, resulting in life-threatening complications. The medical management of ectopic pregnancy has emerged as an important alternative to the surgical one. Surgical treatment of ectopic pregnancy is an invasive method that carries a significant financial burden on healthcare systems and families. It can also be accompanied by serious side effects for pregnant women, such as uterine rupture, sepsis, and death especially when it is performed in unprepared places and not under the supervision of healthcare professionals [26]. The improved safety profile of letrozole usage compared to methotrexate and its successful use to manage different gynecological conditions encourage further research into its use as a medical treatment for ectopic pregnancy [27].

Our study showed that the resolution rate for ectopic pregnancy using a high dose of letrozole (10 mg day^−1^) was 85%, while the resolution rate for a low dose of letrozole of 5 mg day^−1^ was 65%, which was nearly in agreement with a recent study by Mitwally et al. (2020) [27] found that the human resolution rate for ectopic pregnancy using 5 mg day^−1^ of letrozole was 86%. This difference may be attributed to the relatively small number of participants in both studies. On the other hand, these results were in agreement with the experimental study by Tiboni et al. 2009 [7], who reported similar findings in rats treated with letrozole (0.04 mg kg^−1^) equivalent to a 2.5-mg daily human dose.

There was a difference in the resolution rate between the high-dose administered letrozole group GIII 85% and the low-dose administered letrozole group 65%, but statistically, these differences did not appear *P* = 0.144 due to the small number of participants in this study, but the resolution rate of the high-dose group 85% was very close to the ideal results of the control group 100% to the extent that there was no statistical difference between them *P* = 0.230, while the resolution rat of the low-dose group 65% could not get close enough to the optimal results of the control group 100% to dissolve the significant statistical difference between them, where the results between them were statistically significant difference *P* = 0.008*, also, the higher dose letrozole group was able to reduce the β-hCG in a way that is close to the total removal of the ectopic pregnancy by surgery (control group), where there was no statistically significant difference between them *P* = 0.2779., while there was a significant statistical difference between the low-dose group and the control group < 0.001* regarding the reduction of β-hCG.

In this study, the Letrozole-treated group IV showed a significant reduction in ER and PR expression, which play a major role in maintaining pregnancy. Low estrogen levels caused by the aromatase inhibitor letrozole can cause inhibition of progesterone receptors that disrupt the physiological functions of progesterone, which are necessary to maintain early pregnancy. [27].

Vascularization occurs in the human placenta by the formation of new blood vessels from pluripotent precursor cells in the mesenchymal core of the villi, rather than starting from fetal blood cells; the placental villi begin to vascularize on day 21 after conception. [25]. Our results showed that the trophoblastic tissues of the letrozole-treated group IV showed a marked reduction in vascular endothelial growth factor (VEGF) compared to the expression of VEGF in the trophoblastic tissue of the control group. VEGF is a powerful mitogenic agent that promotes the formation of blood vessels in the fetoplacental unit that keeps the fetus alive. [28]. Estrogen is a primary stimulatory factor for VEGF, promoting the development of the vascular network and uterine permeability; therefore, antiestrogenic drugs, such as aromatase inhibitors, are angiogenesis inhibitors and can result in complete failure of fetal expansion by interfering with placenta formation and embryonic vascular development.[13, 29–35]. Additionally, in ectopic pregnancy, maternal cells at the implantation site usually show very limited decidual differentiation, if any [30, 31], which markedly attenuates the secretion of placental growth factor PIGF, in addition to the unfavorable environment represented by hypoxia, added further inhibition of PIGF expression [36]; at the same time, as a compensatory mechanism, ectopic trophoblastic tissue increases VEGF expression more than normal intrauterine conception responding to hypoxia, and other stimulators including estrogen [37–41], which enables it to continue to grow, while tubal implantation that cannot overcome these unfavorable conditions of implantation undergoes spontaneous resolution [42–44]

Apoptosis, or cell death, is a process that occurs naturally during the growth and development of tissues (such as the placenta). Preeclampsia and intrauterine growth restriction are two clinical obstetrics pathologies that have enhanced placental apoptosis [7].

In our study, the placental apoptotic index was measured using the cleaved caspase-3 expression which was found to be higher in trophoblast cells of the letrozole-treated group GIV in comparison to the trophoblastic tissue of the control group that showed lower expression. Caspase-3 is one of the proteases involved in the initiation and execution of apoptosis. Therefore, limiting caspase activity is essential for effective cell survival management.

Investigations of the current study found a considerable increase in liver enzymes (ALT and AST) in the high-dose letrozole-treated group compared to the control and low-dose administered group. These results were experimentally observed by Aydin et al. (2011) [45], who reported an increase in liver function indices in female rats given 1 mg kg^−1^ letrozole. Moreover, up to 1% of women with prolonged letrozole administration have been associated with increased liver enzymes. These increases are generally asymptomatic and self-limited and do not require a dosage modification [27]. Letrozole is an inhibitor of CYP 2C19 and is metabolized by the cytochrome P450 (CYP 3A4 and CYP 2A6) system in the liver; therefore, extreme doses of letrozole can induce liver injury by a toxic or immunogenic metabolite. [46]. The high dose of letrozole 10 mg/day used in our study and 12.5 mg/day were used safely by Pritts et al. (2011) [47] in human patients for induction of ovulation and controlled hyperstimulation of the ovary.

## Conclusion

Letrozole deprives the placenta of estrogen signals, which have a vascular supporting effect, destroying the placental vascular system with marked apoptosis. This study showed that using 10 mg day^−1^ of letrozole resulted in a considerably safe and high success rate of ectopic pregnancy termination without imposing any serious side effects, but even with the higher dose of letrozole, preparation for potential salpingectomy is needed.

## Recommendations and Limitations

The main limitation of this study was the small number of participants; this study draws attention to the necessity of determining the optimal and safe dose of letrozole to achieve the highest success rate in medical termination of ectopic pregnancy, and the results must be confirmed with a large number of participants, and variable studies which facilitate accurate statistical calculations, before being accepted as a standard treatment or a guideline implement.

## Data Availability

Data openly available in a public repository.
